# A Hybrid Process Integrating Reverse Engineering, Pre-Repair Processing, Additive Manufacturing, and Material Testing for Component Remanufacturing

**DOI:** 10.3390/ma12121961

**Published:** 2019-06-18

**Authors:** Xinchang Zhang, Wenyuan Cui, Wei Li, Frank Liou

**Affiliations:** Department of Mechanical and Aerospace Engineering, Missouri University of Science and Technology, Rolla, MO 65409, USA; wcz68@mst.edu (W.C.); wldp5@mst.edu (W.L.); liou@mst.edu (F.L.)

**Keywords:** hybrid process, component remanufacturing, additive manufacturing, reverse engineering, material testing

## Abstract

Metallic components can gain defects such as dents, cracks, wear, heat checks, deformation, etc., that need to be repaired before reinserting into service for extending the lifespan of these parts. In this study, a hybrid process was developed to integrate reverse engineering, pre-repair processing, additive manufacturing, and material testing for the purpose of part remanufacturing. Worn components with varied defects were scanned using a 3D scanner to recreate the three-dimensional models. Pre-repair processing methods which include pre-repair machining and heat-treatment were introduced. Strategies for pre-repair machining of defects including surface impact damage, surface superficial damage and cracking were presented. Pre-repair heat-treatment procedure for H13 tool steel which was widely used in die/mold application was introduced. Repair volume reconstruction methodology was developed to regain the missing geometry on worn parts. The repair volume provides a geometry that should be restored in the additive manufacturing process. A damaged component was repaired using the directed energy deposition process to rebuild the worn geometry. The repaired part was inspected in microstructure and mechanical aspects to evaluate the repair. The hybrid process solved key issues associated with repair, providing a solution for automated metallic component remanufacturing.

## 1. Introduction

Components of jet engines, airfoils, piping systems, heavy duty machines, molds, and dies frequently run in harsh conditions such as extreme heat, rapid heating and cooling, dynamic contact, vibration, overload, severe impact, friction, erosion, fatigue, etc. [1,2]. Therefore, a large number of such valuable parts can be prematurely damaged in types of partial fracture, surface pits, scratches, cracking, peeling, spot corrosion, etc. [3]. Defects such as excessive material cutting may happen during the manufacturing stage due to operator’s error or machine malfunction, resulting in complete discard of the costly products. Due to the expensive raw material, complexity of the parts and high time- and energy-consumption in fabrication, discarding of worn parts is not a wise idea. Fortunately, many options are available in repairing damaged components to maximize their service life by using additive manufacturing (AM) technology.

Electron Beam Melting (EBM) utilizes an electron beam to deposit metal powders on a substrate. This process advances in reduced residual stress as the temperature in the chamber is heated to 700 °C and maintained during the fabrication [4]. EBM makes it possible to acquire excellent material properties, very low porosity and able to join distinct materials together, while a big issue is to create a high vacuum atmosphere (<1 × 10^−4^ bar) [5]. Cold spray bonds filler material with substrate by injecting tiny solid metal particles at supersonic velocities with the aid of heated nitrogen or helium gas stream [6]. Low tensile residual stress exists in deposits due to the relatively low temperature in the process. Major limitations of cold spray include difficulty in coating hard and brittle materials, lack of metallurgical bond and difficulty for depositing on complex surfaces due to limited spray angles. Thermal spray inputs excessive heat to the substrate, causing high residual stress and possible distortion of delicate components [6].

**Figure 1:**
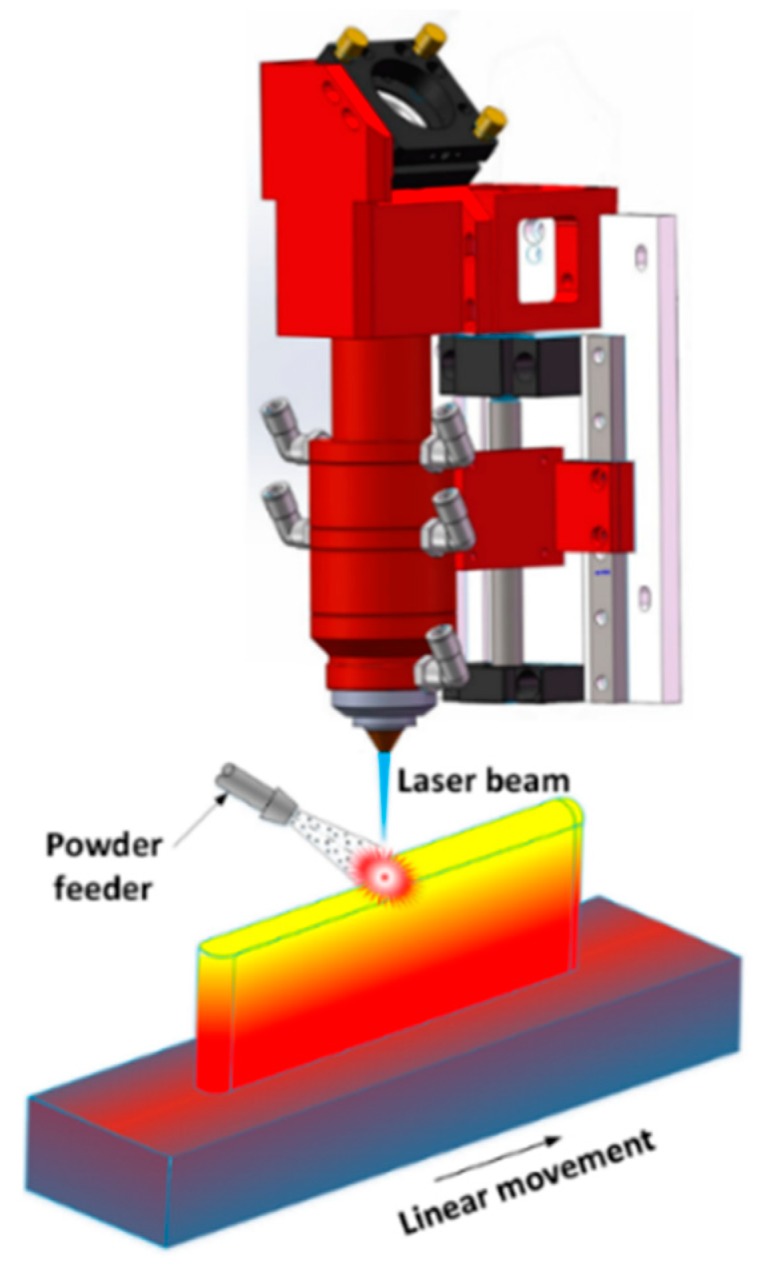
Schematic diagram of the laser-aided direct energy deposition process.

Laser-aided Direct Energy Deposition (DED) (Figure 1) is suitable for part repair. For example, varied shapes of grooves and slots were machined on AISI H13 tool steel substrates and then refilled by depositing H13 tool steel powder [7]. The process can fabricate high-quality repaired parts, but some defects may exist if the defect’s boundary is too steep. Investigators prepared different groove-shaped defects on Stainless Steel and Ti-6Al-4V substrates and attempted to restore these missing volumes by modifying AM processing parameters [8]. The result shows that high quality repaired samples can be obtained with a reasonable combination of processing parameters. Defects on Ti-6Al-4V substrates were also generated and subsequently repaired by filling Ti-6Al-4V powder [9]. The mechanical qualification reveals good bonding strength and higher tensile strength within the deposits than the substrate. In summary, the DED process for repair has the following benefits: (1) less thermal input, which is required for repairing dimensional sensitive parts such as engine blades [10]; (2) good metallurgical bond between deposits and base metals [11]; (3) flexible integration with CNC (computer numerical control) machine or robot to develop a hybrid process to automate the repair [12]. 

Hybrid manufacturing is the process of integrating several processes in a single machine [13]. These processes can be joining, dividing, subtractive, transformative, additive, etc. [14]. The integrated hybrid process provides an ideal industrial solution for complex parts manufacturing or repair. In this way, hybrid manufacturing overcomes the drawbacks of each process by combining another process that has strong corresponding advantages, thus enhancing overall competitiveness. For example, additive manufacturing enables fabrication of freeform structures while associated with low accuracy and coarse surface finish [15]. Subtractive operation such as CNC machining, in contrast, possesses precise production capacity [16]. The combination of both processes takes advantage of each technology, enabling production of end-use products in a single hybrid machine.

Studies have been conducted to utilize the hybrid manufacturing approach to remanufacture worn components. A thin-curved damaged aircraft engine blade was repaired in [10] by depositing material back to the worn area and followed by CNC milling for post-machining. A damaged blade tip was repaired using laser cladding to add materials in the damaged region and the blade was then machined on a CNC machine to obtain the exact dimensions [17]. A hybrid machine was developed to integrate AM with CNC machining that is capable of adding features to existing objects or repairing worn components [18]. 

**Figure 2:**
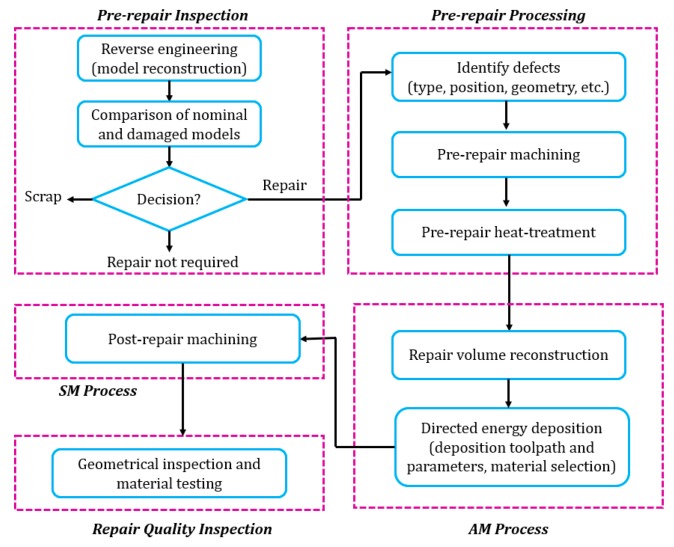
Diagram of the integrated hybrid process.

An integrated hybrid process for component remanufacturing in this study obeys the following procedure as shown in Figure 2: (1) pre-repair inspection; (2) pre-repair processing; (3) AM; (4) subtractive manufacturing (SM); (5) repair quality inspection. In the beginning, a complete inspection was performed on worn parts to assess the feasibility of repair. Considering the excessive variety of locations and geometries of defects, the inspection is highly a case to case basis. After that, pre-repair processing was conducted to guarantee the worn parts were repairable. Previous work in component repair is concentrated on post-machining. However, pre-repair processing is also crucial in the repair chain. Pre-repair processing discussed in this paper includes pre-repair machining and pre-repair heat-treatment. Pre-repair machining is required because many defects such as cracking are not directly accessible to AM systems. Such defects cannot be repaired without machining off materials surrounding the inaccessible defects. The inclination angle of boundaries of defects should also be considered in the machining process since it is reported that too steep walls cannot form sound bonding between filler material and substrates [19]. The AM process builds materials in the damaged area to restore the missing geometry in a layer-upon-layer fashion [20]. The key is to reconstruct the exact missing geometry to provide a tool path for material deposition [21]. SM succeeds the AM process since the as-deposited geometry may have unsatisfied surface roughness. In the quality inspection step, the geometry and mechanical properties of the repaired part were inspected to validate a successful repair.

This paper is organized in the following manner. Section 2 proposes methodologies for pre-repair machining of typical defects including surface impact indentations, surface superficial defects and cracking. Section 3 introduces a pre-repair heat-treatment procedure for H13 tool steel which is widely used in die/mold application. Properties of worn H13 tool steel before and after re-hardening process are compared to validate the benefits of pre-repair heat-treatment. Section 4 introduces a damage reconstruction methodology to recreate the missing geometry on worn parts. Repair experiments and repair quality inspection are conducted in Section 5 to evaluate the remanufacturing process. Reverse engineering is utilized to recreate models of worn components in Section 2 and Section 4. Key conclusions are summarized in Section 6.

## 2. Development of Pre-Repair Machining Strategies 

In general, damaged components cannot be repaired without pre-repair machining. There are several reasons that pre-repair machining is required. At first, it is necessary because the worn area cannot be directly accessed by the AM system, thus melt pool cannot be generated and filler material is not able to be deposited. For example, for repairing cracks on worn parts, it is necessary to machine a slot or groove to remove materials surrounding the cracking area to reveal an accessible area. In addition, worn metal parts usually have heat-checks or damaged layers due to corrosion, erosion, and wear. Such contaminated layers need to be removed before the AM process because direct depositing materials on such regions introduces contamination and thus the bi-material interfacial bonding cannot be guaranteed. Contaminated inclusion between filler material and substrates decimates the mechanical properties of parts and therefore leaves a great threat in service.

The aim of this section is to introduce pre-repair machining methodologies for typical defects. Considering the removed materials must be re-deposited in the AM process, the volume of machined materials should be minimized. The overall methodologies presented in this section are targeted at three types of defects: (1) surface impact defects; (2) surface superficial defects; and (3) cracking.

### 2.1. Pre-Repair Machining Strategy for Surface Impact Defects

**Figure 3:**
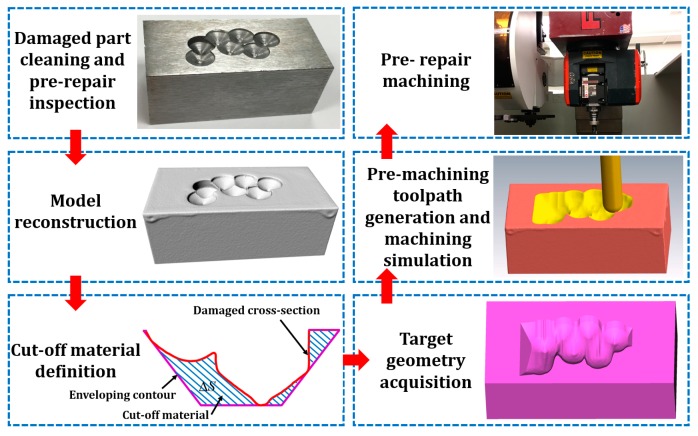
Pre-repair machining procedure for surface impact defects.

The machining procedure for surface impact defects such as dents, notches, grooves and material overcut is illustrated in Figure 3, which includes (1) damaged part cleaning and pre-repair inspection; (2) model reconstruction; (3) cut-off volume definition; (4) target geometry acquisition; (5) toolpath and program generation, and (6) machining. 

#### 2.1.1. Damaged Part Pre-Repair Inspection

**Figure 4:**
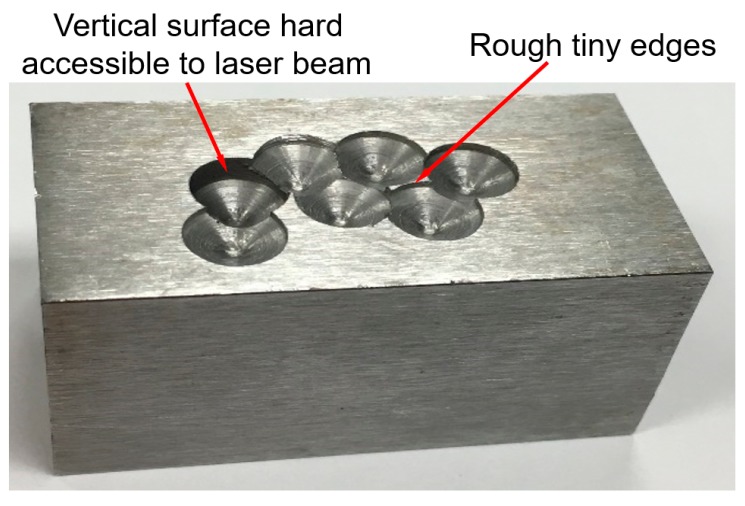
An H13 tool steel substrate with ball-indented defects.

An H13 tool steel block with dimensions of 50 × 25.4 × 25.4 mm^3^, shown in Figure 4, was utilized as an example for illustrating machining strategy for surface impact defects. Ball-indented defects were randomly prepared on the surface of the substrate using an 8 mm drill bit. The holes on the block have varied depths and overlap ratios. 

One can see in Figure 4 that the damaged substrate cannot be repaired directly due to at least two reasons. One is that vertical surfaces on the side of the holes are not accessible to the laser beam and powder feed nozzle for the 3-axis AM system. Therefore, materials surrounding vertical surfaces need to be machined to reveal a tilted surface to guarantee accessibility. Another reason is that the rough tiny edges in the damaged area complicate the toolpath planning for material deposition and may distort during deposition due to heat input. Such deformation could ruin the accuracy of the as-deposited geometry.

#### 2.1.2. Model Reconstruction

**Figure 5:**
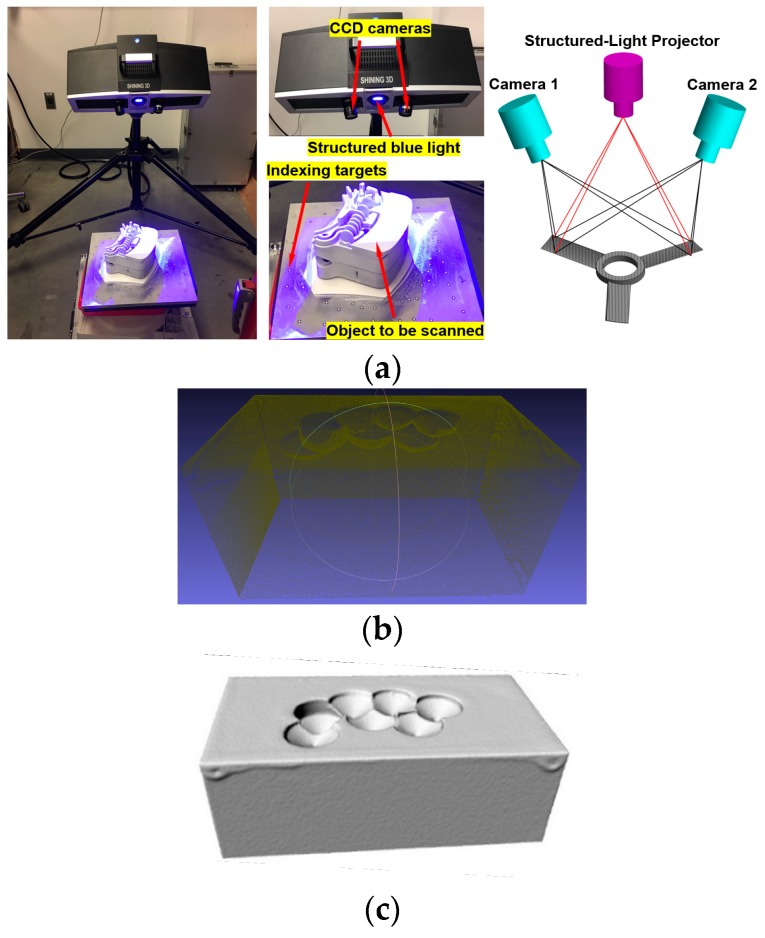
(**a**) 3D scanning setup; (**b**) reconstructed point cloud; (**c**) reconstructed STL model.

Recreating the model of worn part is required for pre-repair machining. In this research, models of damaged parts were reconstructed using a structured-light optical 3D scanner (OptimScan-5M, Figure 5a, Shining 3D, Hangzhou, China). The single scan range of the scanner is 100 × 75 mm^2^. The single shot accuracy of the scanner is 0.005 mm and the volume accuracy is 0.8 mm/m. The scanner has a blue light projector that emits structured-light patterns on an object and two CCD cameras on the scanner measure the distorted dimensions of the pattern. The alteration in dimensions provides the three-dimensional coordinates of the object. In order to acquire a complete model, multiple scans are required, capturing different orientations of the object. For registering point cloud from different scans to a single model, indexing targets need to be randomly pasted around the object to be scanned. Figure 5b depicts the point cloud of the substrate. The point cloud was processed to create the STL (stereolithography) model shown in Figure 5c.

#### 2.1.3. Cut-Off Volume Definition

**Figure 6:**
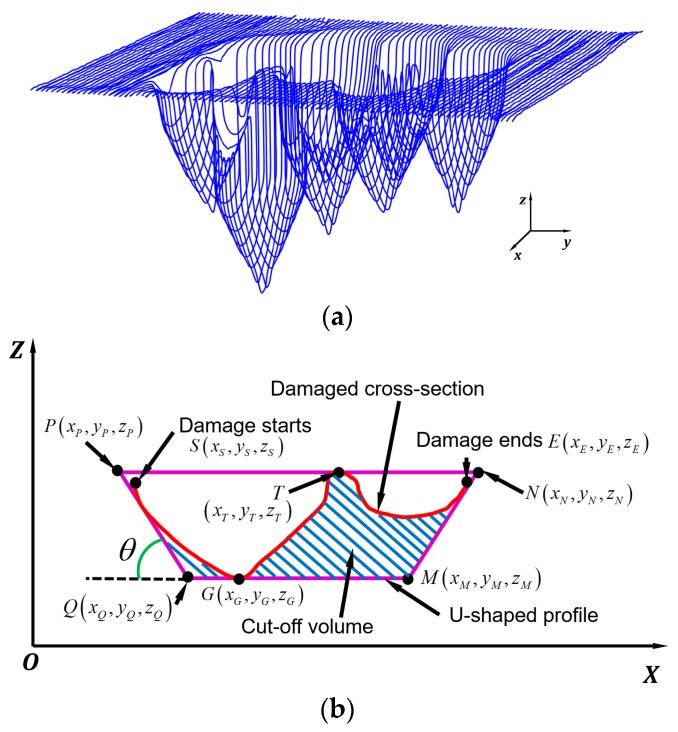
(**a**) Cross-sections of the block; (**b**) U-shaped boundary (UB) definition.

The STL model of the damaged part was sliced into several layers along the y-axis with a layer thickness of 0.5 mm as shown in Figure 6a. The slicing outputs a series of layers that combined damaged and undamaged cross-sections. Damaged cross-sections contain points in damaged and undamaged regions. Damaged points can be extracted by calculating the distance from the points to the nominal surface. Points with distance beyond a tolerance were defined as damaged points. Figure 6b presents a damaged cross-section where damage starts at point *S* and ends at *E*. 

In order to machine off materials around the defects, two enveloping boundaries named U-shaped boundary (UB) and convex-hull boundary (CHB) were utilized. Both profiles contain the damaged area and surrounding materials to provide good accessibility to the AM system.

##### UB Definition

As shown in Figure 6b, two approaching lines *PQ* and *MN* with an inclination angle *θ* were utilized to approach the damaged cross-section. The damage starting point *S* and ending point *E* were known in the damage searching step. It is necessary to find points *P*, *Q*, *M*, and *N* to define the two approaching lines. Such points can be defined as follows: *Z_P_* and *Z_N_* were defined by finding the maximum z-coordinate on the damaged cross-section, which is *Z_T_*. *Z_Q_* and *Z_M_* were defined by exploring the minimum z-coordinate on the damaged cross-section, which is *Z_G_*. Since the damaged model was sliced along the y-axis, *Y_P_* = *Y_Q_* = *Y_M_* = *Y_N_* = *Y_S_* = *Y_E_*. *X_P_* and *X_N_* were randomly defined as long as *X_P_* is less than the minimum x-coordinate of the damaged points and *X_N_* is larger than the maximum x-coordinate of the damaged points. Once *X_P_*, *X_N,_* and the sidewall inclination angle *θ* are defined, the coordinates *X_Q_* and *X_M_* can be calculated according to Equation (1).

(1) $$\left\{ \begin{array}{l} x_{Q}=\frac{z_{P}-z_{Q}}{\tan\theta }+x_{P}\\ x_{M}=x_{N}-\frac{z_{N}-z_{M}}{\tan\theta } \end{array}\right.$$

Once the two approaching lines were defined, the relationship between the approaching lines and the cross-section was checked. If there is no intersection, a step was applied to *X_P_* and *X_N_* to make the lines approach the cross-section, and another checking iteration was conducted. Two lines that firstly have intersections with the damaged cross-section can be obtained and lines before this iteration were gathered, which is shown in Figure 6b.

**Figure 7:**
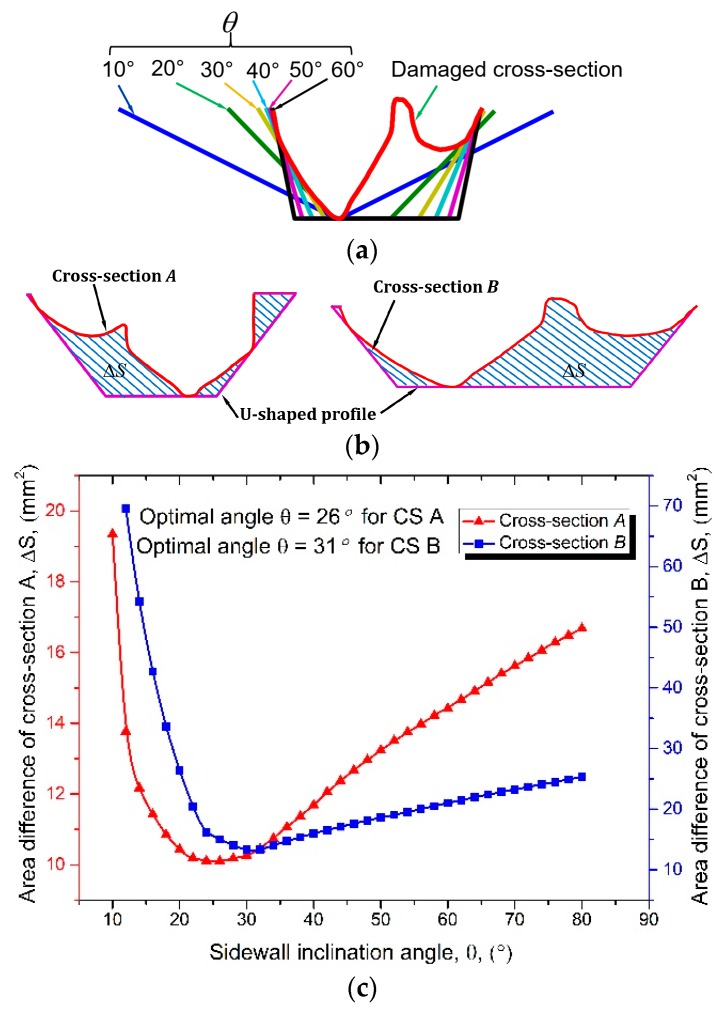
Sidewall inclination angle optimization for UB. (**a**) UB with varied approaching lines; (**b**) UB for cross-sections *A* and *B*; (**c**) relationship between area difference ∆*S* and inclination angle *θ*.

The inclination angle *θ* of the two approaching lines can be operator-determined and adjusted flexibly according to the specifications of the AM system. It should be noted that the inclination angle has a significant effect on the cut-off volume. A small *θ* gives good accessibility but may result in a much greater cut-off volume. An optimized *θ* needs to be determined which not only reveals good accessibility but also holds a minimized cut-off volume. For this purpose, a series of approaching lines with varied inclination angles were adopted to intersect a cross-section shown in Figure 7a. Inclination angle *θ* controls the accessibility and it should not approach 90°. The authors conducted a series of experiments that aimed to refill slots with different sidewall inclination angles and it was revealed that 75° can still yield a sound bi-material bonding and good material properties [20]. Therefore, the range of angle *θ* was limited to 10-75°. The area difference between UB and the cross-section, ∆*S* as shown in Figure 7b, indicates the cut-off area. A series of approaching lines were processed on cross-sections *A* and *B* as shown in Figure 7b and the relationship between ∆*S* and *θ* is plotted in Figure 7c.

It was observed from Figure 7c that for both cross-sections, the area ∆*S* decreases and then increases with the increase of angle *θ*. It was found that the optimal angle *θ* (angle that results in the minimum ∆S) for cross-section *A* is 26° and is 31° for cross-section *B*. The curves indicate that neither a minimum nor a maximum tilt angle can result in the minimum cut-off volume. Therefore, the optimal angle *θ* is highly dependent on the profile of the cross-section and should be determined for each one.

##### CHB Definition

**Figure 8:**
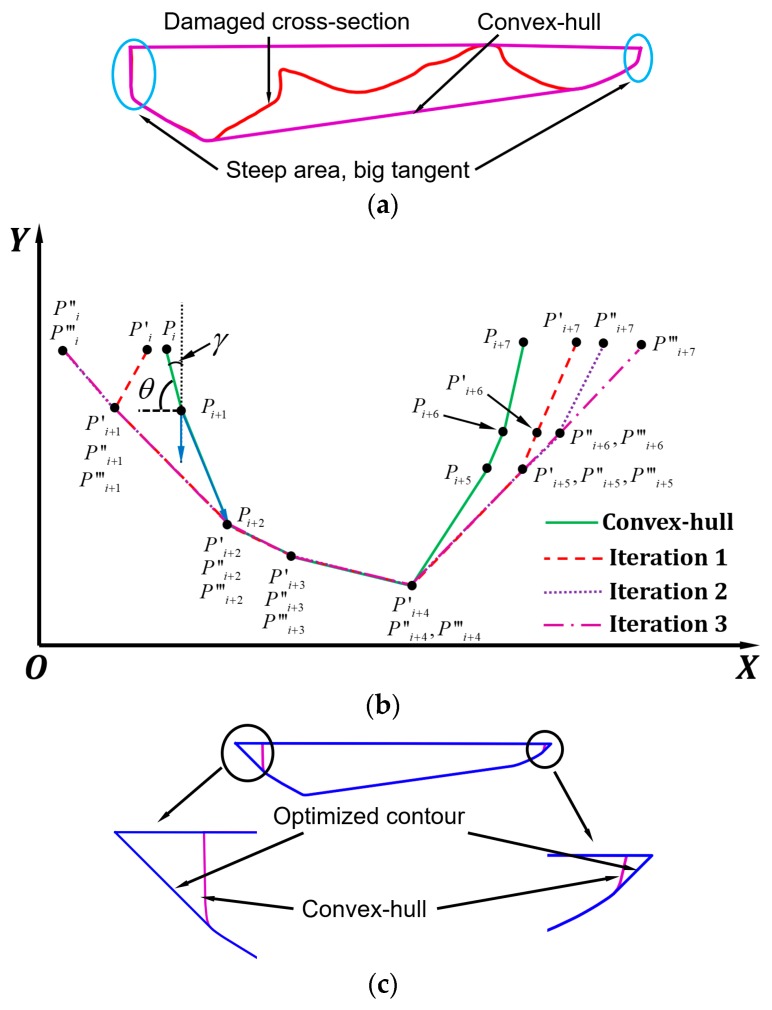
Sidewall inclination angle optimization for the convex-hull boundary (CHB). (**a**) CHB processed on a damaged cross-section; (**b**) schematic diagram showing optimization algorithm; (**c**) optimized contour.

One can see in Figure 6b that for UB, the bottom-line *QM* is parallel to the x-axis, and this may result in extra material cut-off in the polygon *GMETG*. This material over-cut becomes worse when one deep defect is presented while majority defects are shallow. To further minimize cut-off volume, the CHB was obtained as shown in Figure 8a. The convex-hull of any polygon can be easily obtained based on the existing algorithm [22]. However, one should notice that the convex hull cannot be directly utilized as the boundary for machining owing to the possibility of the steep area as shown in Figure 8a. The line segments in such regions have big tangents that cannot guarantee accessibility. Therefore, such lines need to be tilted. The algorithm for tilting lines is schematically depicted in Figure 8b and discussed below.

Suppose polygon *P_i_P_i+1_···P_i+6_P_i+7_* is a convex-hull of one cross-section. The inclination angle of *P_i_P_i+1_* is indicated as *θ*. As *θ* is beyond the allowed angle, *P_i_* needs to be rotated around *P_i+1_* to *P_i_′*. The coordinate of *P_i_′(x_i_′, y_i_′, z_i_′)* can be calculated according to Equation (2), where *(x_i+1_, y_i+1_, z_i+1_)* is the coordinate of *P_i+1_* and *γ* is the complementary angle of *θ*. The inclination angle of *P_i+1_P_i+2_* also exceeds the allowed angle and therefore, *P_i+1_* also needs to rotate around *P_i+2_* to *P′_i+1_*. After that, the polygon is *P′_i_P′_i+1_···P′_i+6_P′_i+7_*. However, the inclination angle *P′_i_P′_i+1_* still surpasses the desired angle and therefore, *P′_i_* needs to be further rotated around *P′_i+1_* to *P″_i_*. After the second iteration, the polygon became *P″_i_P″_i+1_···P″_i+6_P″_i+7_*. There is another iteration to move *P″_i+7_* to *P‴_i+7_*. Finally, the contour *P‴_i_P‴_i+1_···P‴_i+6_P‴_i+7_* is obtained that satisfies the accessibility, and therefore, can be used as the boundary for cut-off volume definition. 

The algorithm was applied to a cross-section as shown in Figure 8a and the optimized contour is depicted in Figure 8c. As illustrated in Figure 8c, the steep area in the original convex-hull was successfully tilted.

(2a) $$\begin{array}{l} \mathrm{when}\;y_{i+1}<y_{i}\;(\mathrm{Decreasing}):\\ {x_{i}}^{\prime }=x_{i+1}-\sqrt{{\left(\frac{y_{i+1}-y_{i}}{\cos\gamma }\right)}^{2}-{\left(y_{i+1}-y_{i}\right)}^{2}}\\ {y_{i}}^{\prime }=y_{i}\\ {z_{i}}^{\prime }=z_{i} \end{array}$$

(2b) $$\begin{array}{l} \mathrm{when}\;y_{i+1}>y_{i}\;(\mathrm{Increasing}):\\ {x_{i+1}}^{\prime }=x_{i}+\sqrt{{\left(\frac{y_{i+1}-y_{i}}{\cos\gamma }\right)}^{2}-{\left(y_{i+1}-y_{i}\right)}^{2}}\\ {y_{i}}^{\prime }=y_{i}\\ {z_{i}}^{\prime }=z_{i} \end{array}$$

#### 2.1.4. Target Geometry Acquisition

**Figure 9:**
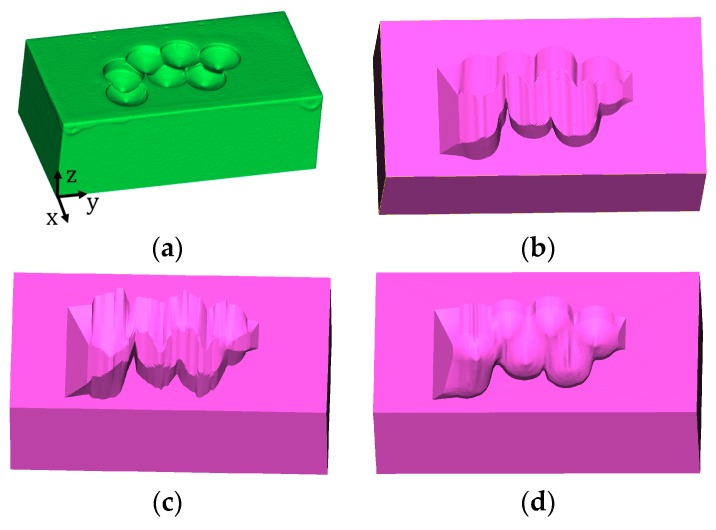
Damaged and machined models. (**a**) Damaged model; model machined using the UB method without optimization (**b**) and with optimization (**c**); (**d**) model machined using the CHB method.

UB and CHB were processed on the model shown in Figure 9a. For UB, sidewall with a fixed angle θ = 45° and optimized angles were both conducted, and the target models are shown in Figure 9b,c, respectively. The model processed using CHB method is presented in Figure 9d.

It can be seen that since the model was sliced along the y-axis, during repair, the tool path should be perpendicular to the y-axis to assure the overall accessibility. If laser moves parallel to y-axis, there might be some regions with poor accessibility. This could be solved by re-slicing the model along the x-axis. However, if the model was sliced along two directions, more material will be removed from the damaged model since each direction requires an amount of material removal. 

The volumes of the damaged model and machined models are summarized in Table 1. It can be observed that UB with the optimized sidewall inclination angle machined less material compared with non-optimized UB. However, CHB results in the least amount of cut-off volume. Therefore, in the machining step, CHB-generated geometry was adopted as the guide model for machining toolpath and program generation.

#### 2.1.5. Machining Toolpath Generation and Machining

**Figure 10:**
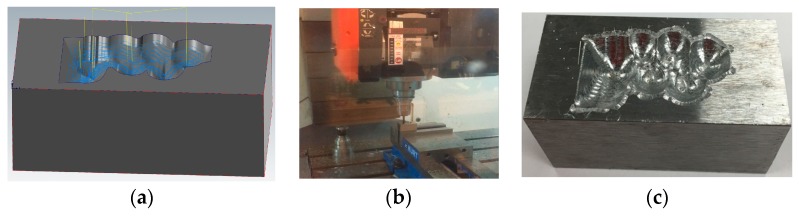
(**a**) Machining toolpath; (**b**) machining setup; (**c**) part after machining.

Once the target geometry was obtained, it was loaded to a CAM (computer aided manufacturing) software (MasterCAM 2018) to generate the machining toolpath as shown in Figure 10a. The milling parameters are listed in Table 2. The damaged part was machined on a Fryer MC-30 with the setup shown in Figure 10b. The machined part is shown in Figure 10c.

### 2.2. Pre-Repair Machining Strategy for Surface Superficial Defects

Casting dies are subjected to rapid heating and cooling cycles during service that causes dimensional distortion and cracking. In addition, frequently contacting with casting alloys such as liquid aluminum causes surface superficial defects such as erosion, wear, and corrosion. It is reported that thermal fatigue and erosion are major contributors to the failure of casting dies [23]. Surface superficial defects can be easily removed by cutting off a thin layer of material from the target surface. The thickness of machined material can be determined once the machined surface is defect-free.

The current research proposed a general procedure for removing surface superficial defects. In general, the damaged component is 3D scanned to recreate the model. After that, the model is loaded to CAM software. The area for machining is defined and machining parameters including cut-off thickness are determined. Subsequently, machining program is generated and transferred to a CNC machine for machining. 

**Figure 11:**
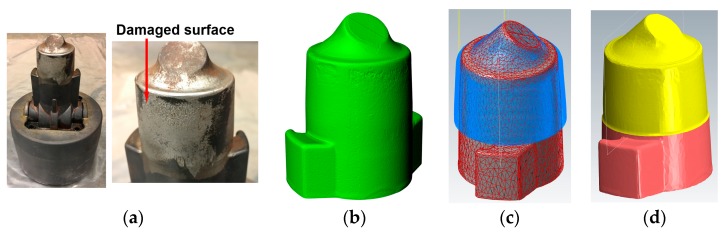
(**a**) A die with surface superficial defects; (**b**) STL model of the die; (**c**) machining toolpath; (**d**) model of the die after machining.

As an example, a casting die shown in Figure 11a has damaged regions on the working convex area that are dominated by tiny rough surfaces. In order to machine off a thin layer of material from the part, the model of the die was generated as shown in Figure 11b. Creating the model of the whole part is not necessary because only the surface with defects needs to be targeted. Generating the whole model not only dramatically slows down the 3D scanning process, especially for a large complex structure, but also makes the toolpath generating process more complicated due to the large size of the imported 3D scanned model.

The volume of materials to be machined depends on the depth of surface defects. The defects of surface erosion, wear and corrosion are usually superficial and can be effectively removed through one pass machining. The cut depth for machining can be determined based on the condition of the damage. For the casting die, materials with 0.5 mm layer thickness were machined. The toolpath was generated as shown in Figure 11c and the simulated machined part is shown in Figure 11d.

### 2.3. Pre-Repair Machining Strategy for Cracking

Heating and cooling cycles during metal casting cause cyclic compressive and tensile stress conditions which lead to thermal fatigue to the casting die [24]. That is why H13 hot work tool steel is usually adopted as die material owing to its high hardenability and good thermal fatigue resistance. Thermal fatigue cracking is a common failure in die-casting dies and engine blades after thousands of shots. The cracking is likely to first appear at corners and edges with small radius [25].

The machining procedure for cracking highly depends on the appearance of cracks and can be determined only after considering a number of factors, such as the depth and length of cracks, surrounding structures, accessibility of cracks to machining tools and AM systems, surface or internal cracking, etc. This paper presents a method for removing cracks on engine blades.

#### Cracking Removal Strategy for Engine Blades

**Figure 12:**
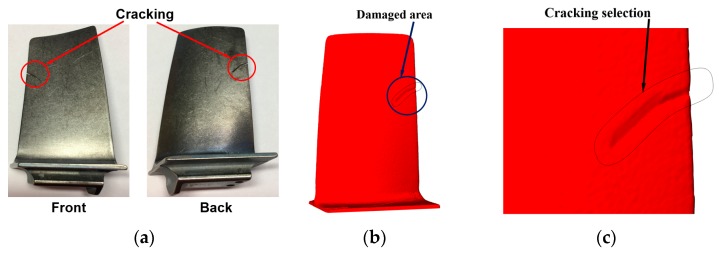

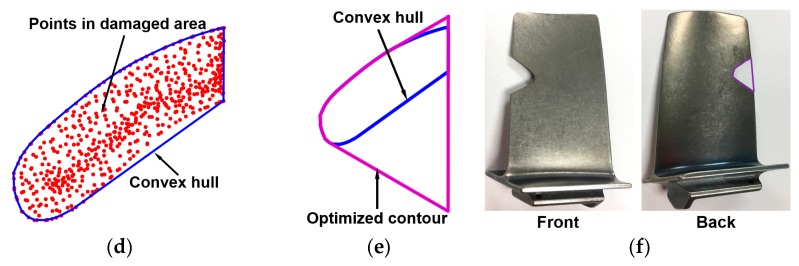
(**a**) Damaged blade; (**b**) 3D model of the blade; (**c**) point cloud in damaged area; (**d**) convex hull of the point cloud; (**e**) optimized contour for machining; (**f**) blade after machining.

Trailing edge cracking is a common failure in turbine or compressor blades mainly due to excessive stress, overloading, overheating or defective materials. Several studies have been targeted at blade repair, mainly focusing on damage extraction [26,27,28], material deposition process planning [29,30,31] and repair automation [17,32], while none report on strategies of pre-repair machining. It is necessary to cut off materials surrounding cracks because non-machined cracks give no accessibility to the AM system, as shown in Figure 12a. This is why the defects in blade edge reported in [21], [17,33] have U- or V-shaped geometries.

To perform machining, the blade shown in Figure 12a was digitally scanned to acquire the 3D model as shown in Figure 12b. After that, the defective area was selected as shown in Figure 12c. By selecting the damaged area, the points located at the worn domain can be easily extracted which is shown in Figure 12d, and subsequently, the convex-hull of the point set was obtained. It should be pointed out that the convex hull cannot be directly used for machining because the bottom portion of the convex hull blocks the accessibility. In order to create a V-shaped geometry, the line segments of the convex hull were tilted according to the algorithm illustrated in Section UB Definition. The maximum sidewall inclination angle is limited to 60°. The finally optimized contour is shown in Figure 12e. EDM wire follows the optimized contour for machining and the machined blade is shown in Figure 12f.

## 3. Development of Pre-Repair Heat-Treatment Procedure 

**Figure 13:**
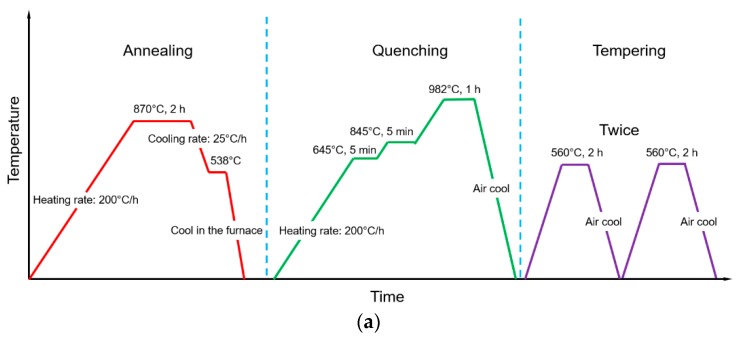

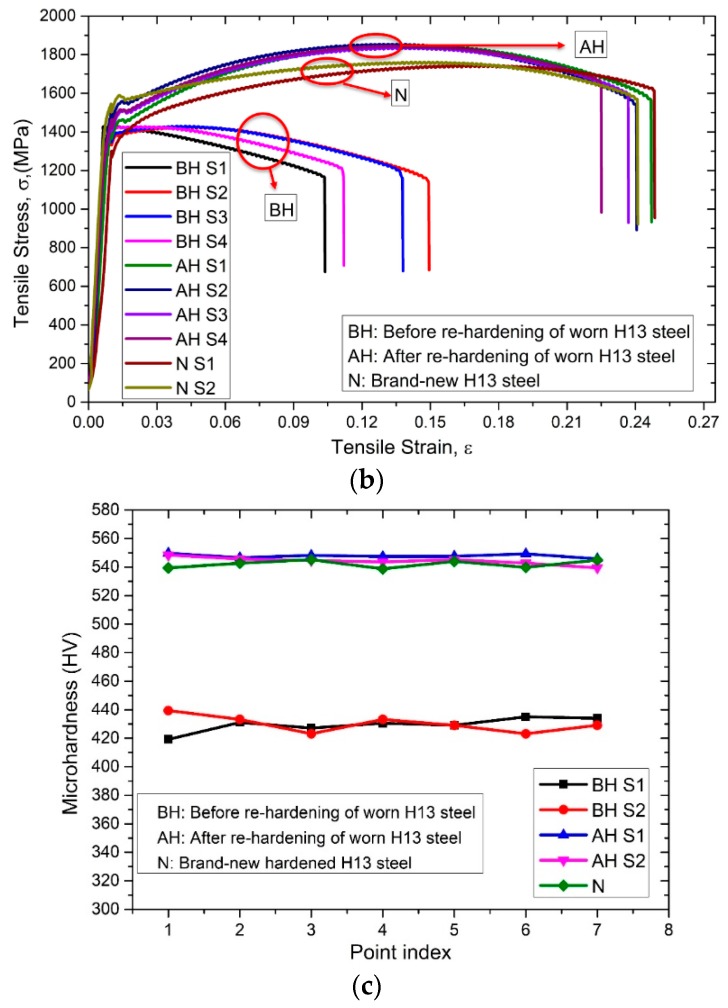
(**a**) Schematic diagram of the re-hardening process for H13 tool steel; (**b**) tensile testing data; (**c**) hardness measurements.

Many parts such as H13 tool steel dies/molds undergo heating and cooling cycles in service and will finally result in thermal fatigue failure. It is reported in [34] that with increasing numbers of thermal cycles, the hardness and microstructure of H13 tool steel change, which eventually results in loss of mechanical strength and plastic deformation. The author reported the hardness of H13 tool steel after thermal fatigue cycles decreased from 650 to 300 HV and the microstructure became coarser and lots of carbides with V and Cr appeared [34]. Without re-hardening, its nominal mechanical properties cannot be restored even if the geometry was repaired. Therefore, pre-repair heat-treatment should be conducted on the worn material before the AM process. Figure 13a depicts the heat-treatment procedure for H13 tool steel. It should be noted that H13 tool steel for die/mold applications is already in the quenched and tempered condition. In order to re-harden it, annealing must be conducted at first, followed by quenching and tempering.

Figure 13b shows the tensile testing data of the worn H13 tool steel (BH), H13 tool steel after re-hardening (AH) and nominal H13 tool steel (N). It can be observed that the Ultimate Tensile Strength (UTS) of BH is 1427.9 MPa. By re-hardening, the UTS can be increased to 1846.7 MPa and the ductility also increased. The tensile properties of re-hardened H13 tool steel are comparable with that of the nominal one. Therefore, it can be concluded that re-hardening restored the tensile property of worn H13 tool steel. 

Figure 13c shows the hardness measurements of BH, AH, and N. In terms of hardness, the BH has a hardness of around 420–440 HV while the AH is about 540–550 HV which is similar to the nominal hardness. The re-hardening process successfully restored hardness of worn H13 tool steel.

## 4. Repair Volume Reconstruction Methodology 

### 4.1. Model Alignment

**Figure 14:**
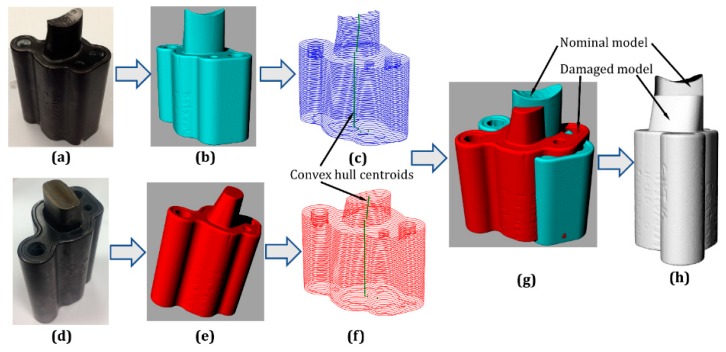
Model alignment. (**a**) Nominal part; (**b**) nominal model; (**c**) cross-sections of the nominal model; (**d**) damaged part; (**e**) damaged model; (**f**) cross-sections of the damaged model; (**g**) models before alignment; (**h**) models after alignment.

As shown in Figure 14, an H13 tool steel die was scanned to recreate the nominal model. After that, defects were created on the part using wire electrical discharge machining (EDM). The damaged component was then captured to recreate the damaged model. Afterward, the damaged model was accurately aligned with the nominal model by using the algorithm discussed in [35] with the following steps.

Step 1: Select an initial slicing vertex *P_0_* and normal direction *n_0_*. The vertex *P_0_* is the point where the slicing process initiates and the normal direction *n_0_* is the slicing direction.
Step 2: Once the initial slicing vertex and direction were selected, a planar reference going through the slicing vertex was utilized to slice the model, generating the cross-section of the layer. After slicing the initial layer, the slicing plane moved according to a defined layer thickness to slice the next layer. The slicing process continues throughout the model. The sliced layers of nominal and damaged models are shown in Figure 14c,f, respectively.
Step 3: The convex-hull centroids of the nominal and damaged models were calculated and obtained as shown in Figure 14c,f.

The model alignment algorithm is to align the convex-hull centroids of the nominal and damaged models. The damaged model can be aligned with the nominal model via three translation values x, y, z and three rotational variables α, β, γ. The transformation matrix combining translation and rotation can be calculated as shown in Equation (3).

(3) $$T=\left[\begin{array}{cccc} \cos\alpha \cos\beta & -\sin\alpha \cos\gamma +\cos\alpha \sin\beta \sin\gamma & \sin\alpha \sin\gamma +\cos\alpha \sin\beta \cos\gamma & x\\ \sin\alpha \cos\beta & \cos\alpha \cos\gamma +\sin\alpha \sin\beta \sin\gamma & -\cos\alpha \sin\gamma +\sin\alpha \sin\beta \cos\gamma & y\\ -\sin\beta & \cos\beta \sin\gamma & \cos\beta \cos\gamma & z\\ 0 & 0 & 0 & 1 \end{array}\right]$$

Suppose $P_{d}=\left(x_{d},\;y_{d},\;z_{d}\right)$ is a point of the convex-hull centroids of the damaged model, the transformed point $P_{d}^{t}=\left(x_{d}^{t},\;y_{d}^{t},\;z_{d}^{t}\right)$ can be obtained from Equation (4).

(4) $$P_{d}^{t}=\left(x_{d}^{t},\;y_{d}^{t},\;z_{d}^{t}\right)=T\times \left[\begin{array}{c} x_{i}\\ \begin{array}{c} y_{i}\\ \begin{array}{c} z_{i}\\ 1 \end{array} \end{array} \end{array}\right]$$

The least squares method was used to acquire x, y, z and α, β, γ. The objective of the least squares method was to minimize the distance of the transformed points $P_{d}^{t}$ to the points of the nominal model $P_{n}$, which is illustrated in Equation (5). The models prior to and after alignment are depicted in Figure 14g,h, respectively.

(5) $$\mathrm{Objective}:\;Min\sum_{0}^{i}{\left(P_{n}-P_{d}^{t}\right)}^{2}$$

### 4.2. Repair Volume Reconstruction

**Figure 15:**
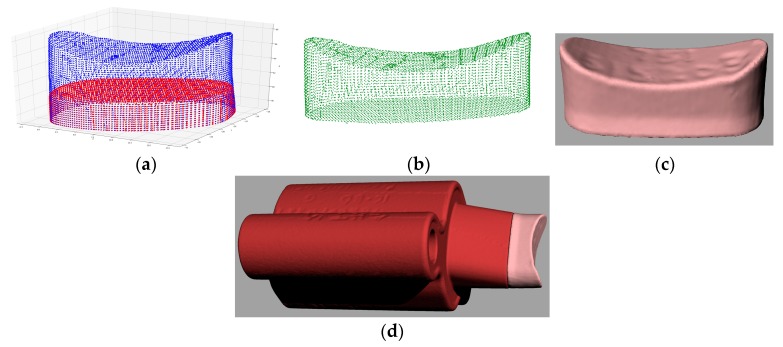
Repair volume reconstruction. (**a**) Intersections of rays with nominal and damaged models; (**b**) extracted points; (**c**) model of the extracted points; (**d**) damaged geometry on the damaged model.

Once the damaged model was aligned with the nominal model, the ray casting method was adopted to regain the repair volume [36]. Casting rays with a specific interval were injected to intersect both the nominal and damaged models, which is depicted in Figure 15a. Intersections of these casting rays with both models can be obtained. By comprising the coordinates of each intersection on the nominal model with the intersection on the damaged model, the point cloud that only belongs to the nominal model and is missing from the damaged model was extracted as shown in Figure 15b. The point cloud was then processed to generate the STL model in Figure 15c. The virtually repaired die is shown in Figure 15d.

## 5. Repair Experiment and Quality Inspection 

### 5.1. Experimental Setup and Material Preparation

The DED system employed in this study consists of a fiber laser with a maximum power of 1 kW (IPG Photonics), blown powder feeder (Bay State Surface Technologies), 3-axis working table and gas feeding unit. Argon gas was used as the powder delivery gas and shielding gas to protect the materials from oxidation. 

The material for repairing the H13 tool steel die in Figure 14 was a Cobalt-based alloy Wallex 40. The reason for selecting this alloy is because Wallex 40 has excellent corrosion and abrasive resistance, and high hardness, thus providing a longer service life of the die. The chemical composition of Wallex 40 and H13 tool steel is listed in Table 3. The processing parameters for repairing the die are summarized in Table 4.

### 5.2. Repair Quality Inspection

#### 5.2.1. Microstructure Characterization

**Figure 16:**
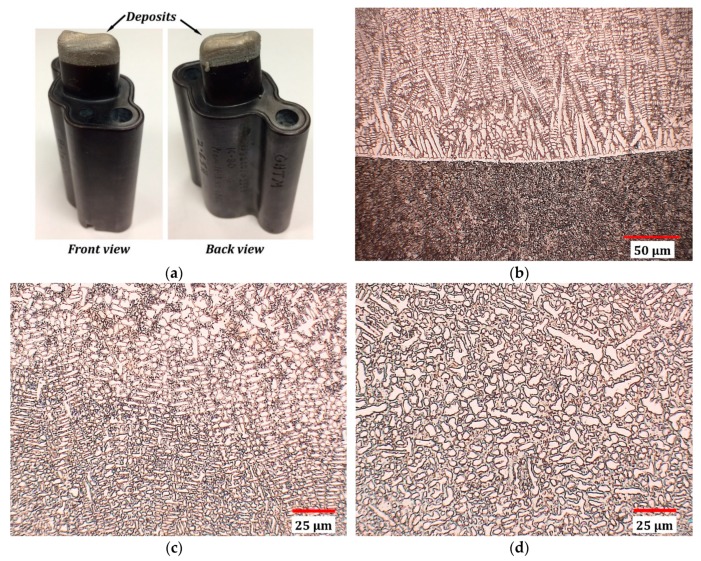
(**a**) Damaged part after repair; optical micrographs of materials at the interface (**b**), in the middle layers of deposits (**c**) and on the top layers of deposits (**d**).

Figure 16a reveals the part after the DED process. It shows the material was successfully restored at the damaged region. In order to evaluate the repair quality, the repair region was sectioned and tested through microstructure characterization and tensile testing.

Figure 16b–d shows the microstructure of materials at the interfacial area (Figure 16b), in the middle layers of deposits (Figure 16c) and on the top layer of deposits (Figure 16d). The microstructure of as-deposited material at the bonding area exhibits a columnar structure which is typical for additively manufactured materials since the cooling rate is high, causing the grains to grow in the heat flux direction. As materials were built layer upon layer, the solidification rate decreased and the deposits changed to dendrite structure with interdendritic eutectics. In the top layers, since the cooling rate is relatively low, the area is composed of mostly equiaxed structure and the quantity of dendrite is small.

It disclosed in Figure 16b that the bi-material interface is distinct. Examination of the area demonstrated the interface is free of defects, confirming the solid bonding between both materials. Besides, no defects were detected at the as-deposited materials, affirming the feasibility of fabricating Wallex 40 using the DED process.

#### 5.2.2. Tensile Properties

**Figure 17:**
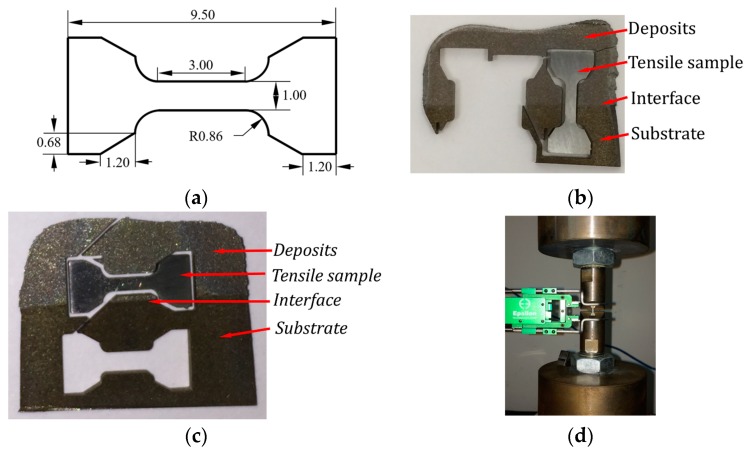
(**a**) Dimensions of tensile specimen (all dimensions in mm, 1 mm in thickness); (**b**) obtained Wallex 40 + H13 tool steel specimen over the sectioned part; (**c**) obtained Wallex 40 specimen over the sectioned part; (**d**) tensile testing setup.

The repaired part in Figure 16a was sectioned using EDM to obtain tensile specimens according to dimensions shown in Figure 17a. Two types of specimens were prepared: (1) Wallex 40 + H13 tool steel specimens with the interface approximately in the middle of the tensile gauge length (Figure 17b); (2) Samples made of as-deposited Wallex 40 (Figure 17c). Samples were tested using an Instron universal tester (Model 5969) with a crosshead rate of 0.015 mm/min. 

**Figure 18:**
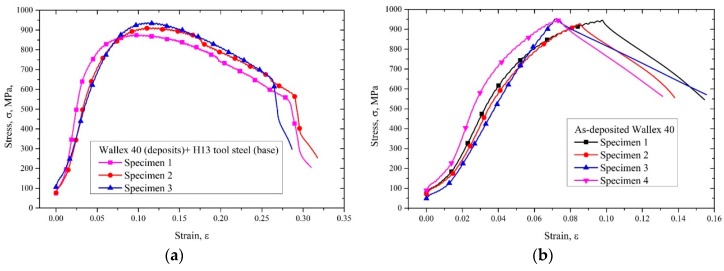
(**a**) Tensile stress–strain curves of Wallex 40 + H13 tool steel specimens; (**b**) tensile stress–strain curves of Wallex 40 specimens.

The tensile stress–strain curves of Wallex 40 + H13 tool steel samples shown in Figure 18 revealed that the tensile stress increased with the increase of tensile strain to a peak of around 900 MPa. Then a yielding region was exhibited before the final fracture, showing the ductile fracture of the samples. The UTS is summarized in Table 5. The average UTS of Wallex 40 + H13 tool steel samples was 908 MPa. The tensile stress–strain curves of Wallex 40 samples increased to about 950 MPa before they suddenly fractured. The average UTS of Wallex 40 was 943.5 MPa.

**Figure 19:**
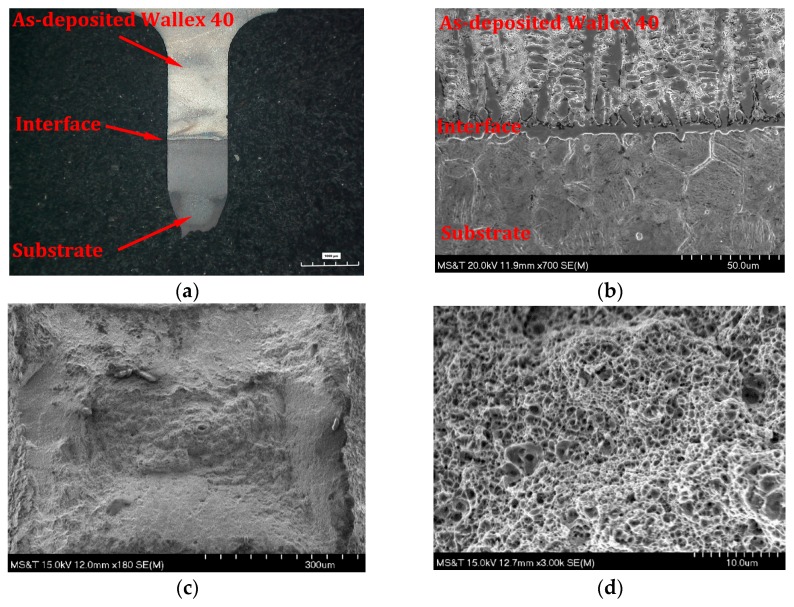
Tensile fracture morphology of Wallex 40 + H13 tool steel sample. (**a**) Overview of the fracture surface in longitudinal section; (**b**) magnified view of the bonding area; (**c**) overview of the fracture surface in cross-section; (**d**) magnified view of the fracture surface in cross-section.

The optical micrograph shown in Figure 19a revealed that the Wallex 40 + H13 tool steel tensile sample fractured at the H13 tool steel region. A necking region was found near the fracture area. The SEM image in Figure 19b shows that the interface is intact and fracture-free, which confirmed the strong adhesion strength. Since the Wallex 40 + H13 tool steel samples fractured at H13 tool steel section, the UTS of as-deposited Wallex 40 is higher than the substrate. This result indicated that the mechanical properties of repaired components were enhanced.

SEM micrographs of the cross-section of the tensile fractured surface are depicted in Figure 19c,d. The overview of the fracture surface revealed unsmooth fracture regions. A large number of voids and dimples were observed in the magnified view revealing the ductile fracture mechanism.

## 6. Conclusions

A hybrid process was investigated to integrate reverse engineering, pre-repair processing, additive manufacturing, and material testing for component repair. The main conclusions are summarized as follows.

- Pre-repair machining methodologies for surface impact defects, surface superficial defects and cracking were proposed. The methods were conducted on worn parts to guarantee the parts were ready for the DED process.
- Pre-repair heat-treatment was performed on damaged H13 tool steel. The result shows the nominal mechanical properties of worn H13 tool steel were successfully restored.
- Repair volume reconstruction strategy based on the ray casting method was introduced and the geometry of repair volume on a worn die was reconstructed.
- Repair experiments were conducted to deposit material in the worn area. Microstructure analysis and tensile testing demonstrated strong bonding along the interface. The UTS of Wallex 40 + H13 tool steel samples is 908 MPa where samples fractured at the H13 tool steel region, while the UTS of Wallex 40 samples is 943.5 MPa.

## Figures and Tables

**Table 1:** The volume of the damaged and machined models.

Model	Damaged	UB, No Optimization	UB, Optimization	CHB
Volume (mm^3^)	22,245.1	21,216.3	21,251.6	21,331.8
Cut-off volume (mm^3^)	-	1028.8	993.5	913.3

**Table 2:** Pre-repair machining parameters for surface impact defects.

Tool	Feed Rate	Spindle Speed	Stepdown	Stepover Percentage	Toolpath Type
0.25″ ball-end	508 in/min	2000 r/min	0.5	33%	Spiral

**Table 3:** Chemical composition of the materials (wt %).

Material	Fe	Co	C	Mn	Si	Cr	Ni	Mo	V	W	B
H13 tool steel	Bal.	-	0.4	0.4	1.0	5.25	-	1.35	1.0	-	-
Wallex 40	1.3	Bal.	0.6	-	1.9	16.2	23.5	-	-	7.6	2.0

**Table 4:** Directed Energy Deposition (DED) processing parameters for repair.

Laser Power	Scan Speed	Powder Feed Rate	Layer Thickness	Track Overlap
800 W	200 mm/min	4 g/min	0.5 mm	0.5

**Table 5:** Ultimate Tensile Strength (UTS) of the repaired samples.

Material	Sample 1	Sample 2	Sample 3	Sample 4	Average	Std.
Wallex 40 + H13 tool steel	875.3	911.4	937.3	-	908.0	31.1
Wallex 40	945.5	927.6	954.5	946.3	943.5	11.33
